# Cerebral microbleed patterns and the risk of incident dementia in elderly adults: The ARIC study

**DOI:** 10.1371/journal.pone.0340361

**Published:** 2026-01-21

**Authors:** Peng Zhang, Huakun Liu, Xiaotan Ji, Jiuchang Zhang, Yuling Cao, Xintan Zhang, Zhongrui Yan

**Affiliations:** 1 Department of Neurology, Jining No.1 People’s Hospital, Jining, Shandong, China; 2 Department of Radiology, Jining No.1 People’s Hospital, Jining, Shandong, China; IRCCS San Raffaele Scientific Research Institute, ITALY

## Abstract

**Objective:**

The association between cerebral microbleeds (CMBs), an indicator of microvascular damage on MRI, and incident dementia (ID) remains inconclusive. We aim to investigate the interplay of CMB presence, number, and location with ID in a large community-based elderly cohort.

**Methods:**

The study included 1532 dementia-free participants (aged ≥ 65 years) derived from baseline examinations (2012–2013) of the Atherosclerosis Risk in Communities Neurocognitive Study (ARIC-NCS). The ID cases were diagnosed at visits 6 (2016–2017), 7 (2018–2019), and 8 (2019–2020). Cox proportional-hazards models were deployed to assess the association of CMB presence, number, and location with ID.

**Results:**

Over a nine-year follow-up (median = 8.1 years), 390 participants developed ID. Microbleed (MB) presence was related to an increased ID risk (HR = 1.24, 95% CI = 1.02–1.51). A stronger association was observed in those with mixed (lobar+subcortical) MBs (HR = 1.60, 95% CI = 1.10–2.30). Compared to participants without MBs, those with ≥ 2 CMBs in any MBs, any lobar MBs, and any subcortical MBs were associated with 57%, 174%, and 74% higher ID risks. Furthermore, those with a pattern of only lobar MBs or superficial siderosis had the highest ID risk (HR = 1.80, 95% CI = 1.26–2.65).

**Interpretation:**

In this large community-based elderly cohort, we identified that the presence of MBs or a high MB count (i.e., ≥ 2), with some specificity for location, was independently related to an increased ID risk over a nine-year follow-up.

## 1. Introduction

Studies of cerebral small vessel disease (CSVD) implication in dementia have largely focused on brain imaging signs, including lacunes, brain atrophy, and white matter hyperintensities (WMHs) [1–4]. Till now, the evidence for the role of cerebral microbleeds (CMBs) in incident dementia (ID) development is limited and controversial. A meta-analysis of five longitudinal studies did not reach statistical significance for the associations between CMBs and ID risk (HR = 1.41, 95% CI 0.90–2.21) [5]. However, two additional population-based studies have provided further insight regarding the relationship between CMBs and dementia risk. These studies all demonstrated an elevated risk of dementia ranging from 70% to 90% for individuals with microbleeds (MBs) [6,7]. Furthermore, a longitudinal study indicated that individuals with ≥ 3 CMBs showed a more rapid deterioration in processing speed, memory, and global cognitive function [8]. Collectively, the evidence remains inconclusive regarding the role of CMBs in ID pathogenesis, as the majority of studies to date have been constrained by small sample sizes and limited statistical power [9–13] or short follow-up periods [6,8,12,13]. It is imperative to address the latter, as MB progresses slowly and participants manifest minimal to no cognitive change over shorter follow-up periods. Furthermore, to our knowledge, limited research has delved into the link between CMBs and ID, specifically in elderly community-dwelling participants. Consequently, there is a compelling need to conduct a comprehensive investigation into the relationships between CMBs and ID risk in a large sample size of community-dwelling older adults.

Depending on their locations, CMBs play roles in both neurodegenerative-related and cerebrovascular-specific pathology in dementia development [7]. Lobar MBs are more likely to be associated with cerebral amyloid angiopathy (CAA), a condition regarded as neurodegenerative-related pathology. Subcortical MBs, especially in deep gray and white matter, tend to be linked with hypertensive vasculopathy and, thus, are considered vascular indicators [14]. Consequently, MBs may offer an intriguing link between neurodegenerative and cerebrovascular pathological mechanisms in the development of dementia. However, it is inaccurate to distinguish the type of brain tissue lesion merely on the basis of the anatomical location of CMBs. For instance, in the case of strictly lobar MBs in elderly individuals, there is strong diagnostic precision for CAA [15], but other pathologies can occur [16,17]. Superficial siderosis (SS), an imaging marker of small vessel disease, is characterized by hemosiderin deposition in or overlying the superficial cortex [18] and, when evaluated in combination with strictly lobar MBs, provides better diagnostic accuracy for CAA [19]. Likewise, individuals who combined subcortical MBs or mixed MBs with superficial siderosis are more likely to be associated with hypertensive vasculopathy [19]. Therefore, we hypothesized that the combination of CMBs and SS may be an optimal means for identifying participants at a high risk of dementia.

Using the Atherosclerosis Risk in Communities Study (ARIC) data, the present study aims to investigate whether the presence, number, and location of CMBs are associated with an escalated ID risk in community-dwelling older adults. This prospective cohort, characterized by large samples (≥ 1500 participants) and long-term follow-up (≥ nine years), can address limitations persisting in prior approaches. Additionally, we further explored the mixed contribution of CMBs and SS in the relationship with ID.

## 2. Method

### 2.1. Study population

The ARIC is a large community-based prospective cohort with 15,792 participants (45–64 years) recruited between 1986 and 1990 from four United States communities [20]. The ARIC Neurocognitive Study (ARIC-NCS) was conducted as part of the ARIC visit 5, which is regarded as the baseline visit for our study. A total of 1980 participants (aged 66–90 years) who underwent brain MRI and neurocognitive testing were selected for baseline analysis [21]. Follow-up neurocognitive tests were conducted at visits 6 (2016–2017), 7 (2018–2019), and 8 (2019–2020). S1 Fig presents further study details. In total, 1,532 subjects were eligible for the final analyses. All participants signed informed consent, and the study was authorized by the Institutional Review Boards at each study center.

### 2.2. Brain MRI and microbleed quantification

As previously delineated, structural brain images were obtained at each field center using 3-Tesla scanners [22]. CMBs were defined as hypointense homogenous lesions (generally 2–5 mm or sometimes 10 mm in diameter) using a T2*GRE sequence [18]. Depending on the anatomical location, CMBs were categorized as lobar (at lobar or cortical gray; including frontal, occipital, temporal, parietal, and insula) and subcortical (at subcortical or periventricular; including thalamus, basal ganglia, corpus callosum, internal capsule, and deep and periventricular white matter) MBs [21–23]. SS was identified as hypointensity on the T2*GRE sequence that followed the contour of the gyrus or sulci [18]. WMH volumes were calculated by a semiautomated segmentation algorithm [14]. Lacunes were assessed visually on fluid-attenuated inversion recovery/T1-weighted MRI scans [23]. Using the Freesurfer atlas, we additionally calculated the volume of the hippocampus and other Alzheimer’s disease signature regions (cuneus, precuneus, entorhinal, inferior parietal lobule, and parahippocampal) [14].

### 2.3. Incident Dementia

Dementia cases of participants who participated in the in-person follow-ups (at visit 5 NCS, 6, 7, and 8) were adjudicated by expert committee review using comprehensive neurocognitive test batteries [24–26]. For participants who died or declined in-person follow-up waves, ID was identified from dementia surveillance using hospitalization and death certificate codes and annual follow-up telephone interviews [23–25,27]. The onset date of ID was determined by identifying the earliest occurrence among the following events: the first follow-up visit when dementia was identified, the first telephone interview with the participant or proxy with the diagnosis of dementia, and the first hospital discharge or death certificate codes for dementia [23].

### 2.4. Covariates

In the present study, we incorporated potential confounders that had previously been considered to be associated with dementia, including sociodemographic, lifestyle, and genetic factors [28]. Sociodemographic information obtained at the study baseline included self-reported sex (male and female), race (white and black), and educational level (i.e., < high school, high school, ≥ college). As MBs were evaluated at visit 5, other covariates were defined at this visit unless otherwise indicated: age, body mass index (BMI; weight/height^2^), apolipoprotein E (*APOE*) ε4 genotype (ε4 carriers versus noncarriers), smoking status (ever vesus never), low-/high-density lipoprotein (LDL)/(HDL), diabetes (defined as hemoglobin A1c ≥ 6.5%, fasting glucose ≥ 126 mg/dL, non-fasting glucose ≥ 200 mg/dL, physician-confirmed self-reported diagnosis, or usage of insulin or diabetes drugs), hypertension (defined as using hypertension medication, or SBP ≥ 140 mmHg and/or DBP ≥ 90 mmHg), and depressive symptoms (evaluated by the 11-item Center for Epidemiologic Studies Depression Scale with a threshold score of 9 or higher).

### 2.5. Statistical analysis

The participants’ characteristics were compared across three MB categories (no MBs, 1 MB, and ≥ 2 MBs) using the chi-square test for categorical variables and the one-way ANOVA or the Kruskal-Wallis test for continuous variables. Descriptive statistics were also employed to compare baseline characteristics between subjects with and without ID.

The evaluation of CMB patterns was conducted by initially analyzing each location independently (lobar: yes/no; subcortical: yes/no). Subsequently, a variable was developed to represent the overall pattern based on MB location and distribution. This variable included the following categories: none (used as the reference), only lobar MBs, only subcortical MBs, and mixed (lobar+subcortical) MBs [19]. We further evaluated these categories, incorporating SS alongside lobar MBs as a potential indicator of CAA (considering no MBs or superficial siderosis as the reference, versus only lobar MBs or superficial siderosis, only subcortical MBs and mixed (subcortical MBs+either lobar MBs or SS)) [19]. Cumulative incidences of ID were plotted using Kaplan-Meier curves for the different MB patterns. Statistical differences were assessed through log-rank tests. Cox-proportional hazard models were deployed to investigate the effect of the presence (no; yes), number (0; 1; ≥ 2), and pattern of MBs evaluated in association with ID. We constructed three models for the Cox-proportional hazards analyses. Model 1 was adjusted for age, sex, race, BMI, depressive symptoms, *APOE* ε4 allele, and educational level. Model 2 was additionally adjusted for ever smoking, HDL-C, LDL-C, hypertension, and diabetes. Model 3 was adjusted for Model 2 plus hippocampal volume, an imaging marker for neurodegenerative processes.

Furthermore, we employed multiplicative interaction analyses to examine the potential modification effects of covariates and MBs. When statistical interactions were identified, additional stratified analyses were conducted to assess the direction and magnitude of the interaction.

Three sets of sensitivity analyses were also employed to ascertain the findings’ robustness. First, we excluded participants with mild cognitive impairment to eliminate the potential effects of reverse causation. Second, we excluded participants whose ID occurred within two years of follow-up. Third, in order to test the associations independent of genetic influences, we reanalyzed the correlation between MBs and ID by excluding participants with *APOE* ε4 status.

Additionally, the improvement in the presence of ID risk reclassification and discrimination with CMBs was evaluated by computing integrated discrimination index (IDI) and net reclassification improvement (NRI). The IDI is employed to quantify the increase in the separation of events and nonevents, while the NRI is used to quantify the amount of correct reclassification introduced by using a model with added variables. The covariates involved in the baseline model are the same covariates used in Cox-proportional hazard analyses. When the NRI or IDI > 0 [29,30], it indicates that the new model shows a performance improvement compared to the old models. In addition, the predictive power of the new models is shown to be better when the calculated NRI or IDI values are larger.

All statistical analyses were performed utilizing R (v4.2.0) and EmpowerStats (X&Y Solutions, Inc., Boston, MA). Results with p < 0.05 were deemed statistically significant.

### 2.6. Ethics approval and consent to participate

The study followed the Declaration of Helsinki and was authorized by the Ethics Committee of Jining No.1 People’s Hospital (2024-IIT-069).

## 3. Results

### 3.1. Participants characteristics

Of the 1532 participants, the mean age was 76.1 years, 40.3% were male, and 25.9% were black. During the 9-year follow-up (median, 8.1 years; interquartile range, 6.8–8.7), 390 participants were diagnosed with ID. Compared to dementia-free individuals, those with dementia were more likely to be older, obese, less educated, show worse cognitive performance, and have worse depressive symptoms. Furthermore, individuals who developed dementia have a higher prevalence of the *APOE* ε4 allele and a higher burden of MRI markers (including higher volume of the hippocampus, nonhippocampal AD signature region, and white matter hyperintensities and higher prevalence of lacunar infarcts and presence of MBs) (S1 Table). Additionally, compared to individuals with no MBs, those with MBs were slightly older, more likely to be male, had an escalated burden of white matter hyperintensities, and had higher lacunar infarct prevalence (S2 Table).

### 3.2. Association between the presence, number, and location of MBs with ID

**Fig 1:**
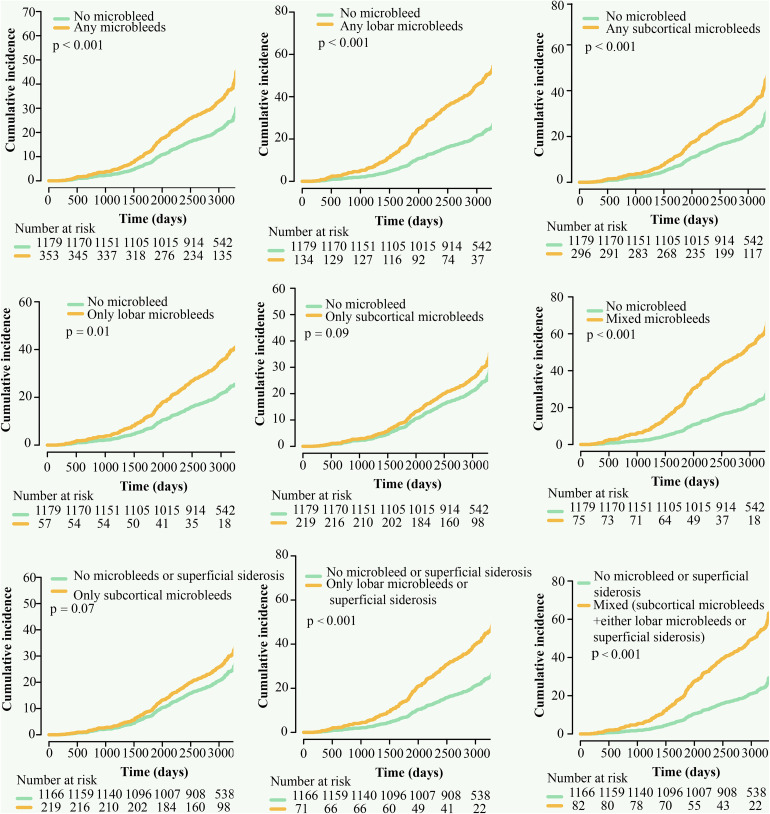
Cumulative risk of incident dementia, by microbleed patterns.

**Fig 2:**
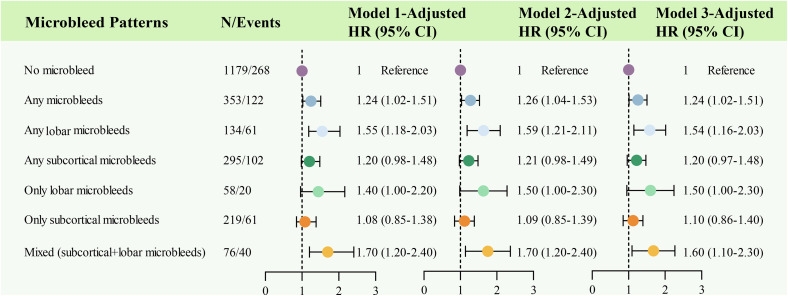
Cerebral microbleed patterns and the risk of incident dementia. The figure summarizes Cox proportional hazards analyses for studying associations between cerebral microbleed patterns and incident dementia. Model 1 was adjusted for age, sex, race, body mass index, depressive symptoms, *APOE* ε4 allele, and educational level. Model 2 was additionally adjusted for ever smoking, HDL-C, LDL-C, hypertension, and diabetes. Model 3 was adjusted for Model 2 plus hippocampal volume.

Cumulative incidence curves (Fig 1) showed that, compared to participants with no MBs, those with any MBs, any lobar MBs, any subcortical MBs, only lobar MBs, and mixed (lobar+subcortical) MBs had a higher risk for ID. After adjustment for age, sex, race, BMI, depressive symptoms, *APOE* ε4 allele, and educational level, any MBs (HR = 1.24, 95% CI 1.02–1.51), any lobar MBs (HR = 1.55, 95% CI 1.18–2.03) and mixed (lobar+subcortical) MBs (HR = 1.70, 95% CI 1.20–2.40) were significantly connected with ID (Fig 2, Model 1). When additionally adjusting for ever smoking, HDL, LDL, hypertension, and diabetes at baseline (Fig 2, Model 2), the above three MBs patterns were still independently associated with a higher risk of ID (HR = 1.26, 95% CI 1.04–1.53; HR = 1.59, 95% CI 1.21–2.11; HR = 1.70, 95% CI 1.20–2.40). After finally adjusting for hippocampal volume (Fig 2, Model 3), a significant association was still recognized for the presence of mixed (lobar+subcortical) MBs (HR = 1.60, 95% CI 1.10–2.30), stronger than for the presence of any MBs (HR = 1.24, 95% CI 1.02–1.51) and presence of any lobar MBs (HR = 1.54, 95% CI 1.16–2.03).

**Fig 3:**
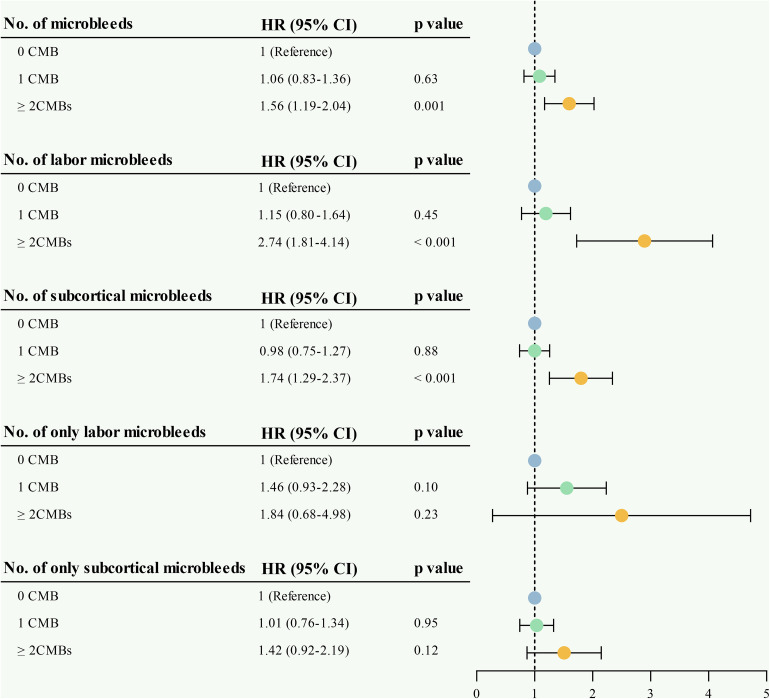
Associations between the location and number of cerebral microbleeds and incident dementia. The figure summarizes Cox proportional hazards analyses for studying associations between the location and number of cerebral microbleeds with incident dementia. Model 1 was adjusted for age, sex, race, body mass index, depressive symptoms, *APOE* ε4 allele, and educational level. Model 2 was additionally adjusted for ever smoking, HDL-C, LDL-C, hypertension, and diabetes. Model 3 was adjusted for Model 2 plus hippocampal volume.

In accordance, compared to participants with no MBs, those with ≥ 2 MBs had a significant association with ID (HR = 1.56, 95% CI 1.19–2.04). There were consistent association patterns for individuals with ≥ 2 any lobar or subcortical MBs (HR = 2.74, 95% CI 1.81–4.14; HR = 1.74, 95% CI 1.29–2.37). However, only lobar or subcortical MBs showed no significant associations (HR = 1.84, 95% CI 0.68–4.98; HR = 1.42, 95% CI 0.92–2.19, Fig 3).

**Fig 4:**
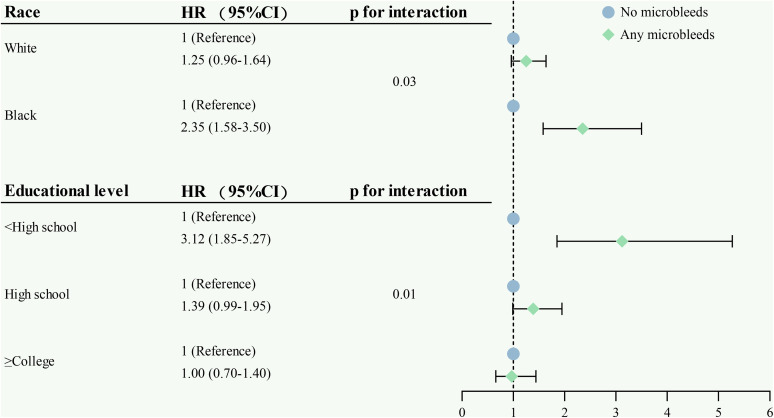
Forest plot of the associations of cerebral microbleeds with incident dementia by race^a^ and educational level^b^. ^a^Hazard ratio (95% confidence interval) was adjusted for age, sex, body mass index, depressive symptoms, *APOE* ε4 allele, educational level, ever smoking, HDL-C, LDL-C, hypertension, diabetes, and hippocampal volume. ^b^Hazard ratio (95% confidence interval) was adjusted for age, sex, race, body mass index, depressive symptoms, *APOE* ε4 allele, ever smoking, HDL-C, LDL-C, hypertension, diabetes, and hippocampal volume.

In the total population, we found a statistically significant interaction of race and educational level with any MBs on ID (p for interaction = 0.03 and 0.01, respectively). Further stratification analysis indicated that the associations of any MBs with ID were stronger in black and low levels of education than in white and high levels of education (Fig 4).

### 3.3. Associations of MB location pattern combined SS with ID

Additionally, we combined MB location with SS, a CAA-type marker, to evaluate the mixed contribution to the development of ID. A similar association was observed for the mixed pattern (subcortical MBs+either lobar MBs or SS) of CMBs (HR = 1.44, 95% CI 1.02–2.05), independent of common dementia risk factors (Table 1). Furthermore, compared to participants with no MBs or SS, those with a pattern of only lobar MBs or SS displayed the highest ID risk (HR = 1.80, 95% CI 1.26–2.65). Similar to the previous result, no significant association was observed between only subcortical MBs and ID (HR = 1.11, 95% CI 0.87–1.42). Cumulative incidence curves showcased that, compared to individuals with no MBs or SS, those with only lobar MBs or SS and mixed (subcortical MBs+either lobar MBs or SS) pattern of CMBs had a higher risk for ID (Fig 1).

**Table 1:** Associations between microbleed patterns, superficial siderosis, and incident dementia.

	Incident Dementia: Model 1			Incident Dementia: Model 2			Incident Dementia: Model 3		
Microbleeds Characteristic	HR	95% CI	p	HR	95% CI	p	HR	95% CI	p
No MBs or SS	1	reference		1	reference		1	reference	
Only subcortical MBs	1.10	0.86-1.40	0.46	1.11	0.87-1.41	0.41	1.11	0.87-1.42	0.39
Only lobar MBs or SS	1.75	1.24-2.48	0.002	1.87	1.32-2.66	<0.001	1.80	1.26-2.65	0.001
Mixed (subcortical MBs + either lobar MBs or SS)	1.48	1.05-2.08	0.03	1.48	1.05-2.10	0.03	1.44	1.02-2.05	0.04

Abbreviations: MBs = microbleeds; HR = hazard ratio; CI = confidence interval; SS = superficial siderosis.

The table summarizes Cox proportional hazards analyses for studying associations between microbleed patterns, superficial siderosis, and incident dementia. Model 1 was adjusted for age, sex, race, body mass index, depressive symptoms, *APOE* ε4 allele, and educational level. Model 2 was additionally adjusted for ever smoking, HDL-C, LDL-C, hypertension, and diabetes. Model 3 was additionally adjusted for baseline hippocampal volume.

### 3.4. Sensitivity analysis

First, we reanalyzed our data after excluding participants diagnosed with mild cognitive impairment at the baseline. In comparison with no MBs, the presence of any MBs, any lobar MBs, and mixed (subcortical+lobar) MBs had a higher risk of ID, whereas only lobar MBs and only subcortical MBs remained non-significant. Second, the primary findings did not change significantly after excluding participants whose dementia was diagnosed in the first two years of follow-up. Third, when the subjects were limited to negative *APOE* ε4 carrier status, the effects of the above pattern of MBs on the risk of dementia were also sustained (S3 Table). Additionally, in the sensitivity analysis, similar associations were observed when we combined MB location with superficial siderosis (S4 Table).

### 3.5. Improvement in the prediction model for ID risks by the addition of the MB patterns

We compared the performance of different models for predicting the ID risks in participants of the ARIC study (S5 Table). The addition of the presence of MBs to the basic model improved the performance validated by IDI. Surprisingly, the NRIs of the MB patterns (any MBs, any lobar MBs, any subcortical MBs, only lobar MBs, only subcortical MBs, and mixed MBs) for estimating ID risks were 17.00% (95% CI = 8.40–24.40), 16.20% (7.10–25.40), 17.20% (8.30–24.30), 17.80% (8.60–26.50), 17.20% (9.70–26.00), and 15.60% (6.70–23.50), respectively. To sum up, the addition of the MB patterns to the basic model with traditional risk factors improved the predictive ability for ID risks, as validated by the IDI and NRI (all p < 0.001).

## 4. Discussion

In this large community-based elderly cohort, we identified that the presence of MBs or a high MB count (i.e., ≥ 2), with some specificity for location, was independently associated with an increased risk of ID over a 9-year follow-up. In addition, a stronger association was observed in participants with a pattern of only lobar MBs or superficial siderosis for dementia. Collectively, our findings provide amplified evidence that MBs function as an independent contributor to ID risk and highlight the need to consider superficial siderosis alongside MBs.

Our results align with other studies [6,7], which indicated that the presence of MBs was related to a statistically significant increased ID risk. A population-based study of over three thousand participants indicated that the presence of MBs was related to a twofold increased risk of ID over a mean follow-up of 4.8 years [6], while another study reported a 74% elevated ID risk over 6 years [7]. Conversely, other studies have suggested no link between MBs and the risk of dementia [12,13]. These inconsistent results could stem from reverse causation bias due to short follow-up periods in previous studies (i.e., < 5 years). The prolonged duration of the follow-up period, which extends up to 9 years, serves to mitigate the potential for reverse causation bias in our study. Moreover, the majority of previous studies did not adjust for hippocampal volume, a significant neurodegenerative confounder in ID [6,8–13]. Consequently, it is possible that ongoing neurodegeneration accounted for the onset of dementia shortly after the initial assessment. Therefore, our present study augmented the clinical significance of MBs. Furthermore, our findings demonstrated that MB severity (the number of MBs) at baseline was also linked to an elevated risk of ID. Previously, several longitudinal studies have indicated relationships between multiple MBs and cognitive decline [6,31,32]. However, these studies either had a lower mean age or did not evaluate this association separately for different MB locations. The present findings, thus, contribute substantially to our understanding of the cognitive consequences of MB severity in community-dwelling elderly individuals.

It was determined that the relationships with ID exhibited variation following the spatial location of CMBs. In our present study, a statistically marginal association was detected between MBs in strictly lobar regions, suggestive of CAA, and the risk of ID. However, the result became significant when we linked strictly lobar MBs and superficial siderosis together to evaluate the risk of ID, which was consistent with a recently published study that the lobar-only pattern of MBs or superficial siderosis was most significantly correlated with cerebral amyloid-beta deposition [19]. Furthermore, no elevated risk was observed for any subcortical MB patterns in conjunction with ID, indicating that merely hypertensive vasculopathy has a relatively weaker effect on the development of ID. Notably, we found that mixed (lobar+subcortical) MBs were most strongly correlated with the risk of dementia. Our results emphasize the role of amyloid pathologic features and cerebrovascular-specific pathology in the pathogenesis of dementia. However, the mechanism of amyloid pathologic features interacting with cerebrovascular-specific pathology to trigger dementia remains unclear. Two relatively well-studied mechanisms are discussed: First, vascular cell APOE4 leads to tight junction impairments, increased pericyte migration and degeneration, decreased synaptic plasticity, reduced blood flow, and increased Aβ deposition in the form of CAA [33]. Additionally, cerebrovascular deposition of Aβ has been demonstrated to impair the reactivity of microvasculature, leading to subsequent microstructural changes of cerebrovasculature and the development of hemorrhagic brain lesions [34–38]. Second, hypertension-associated CSVD also leads to dysfunctions in amyloid clearance and further enhances the accumulation of amyloid deposition in vessel walls [39–41].

Interestingly, we detected potential interactions of any MBs with race and educational level on ID such that the relationships of any MBs with the development of dementia were stronger in black and low levels of education than in white and high levels of education. In keeping with this finding, previous research has reported that black individuals have limited access to education, which may lead to socioeconomic disparities and earlier differences in cognitive reserve. Furthermore, their limited access to healthcare services may contribute to an escalated burden of vascular risk factors [42,43]. Therefore, the reasons above might partially account for the race and educational level-specific differences in the correlation of MBs with the risk of dementia.

The advantages of our study are the longitudinal community-based design with a large sample size, the long follow-up times, the comprehensive cognitive assessments, and the standardized dementia detection system. However, several limitations exist in our study. First, employing the T2*GRE sequence, rather than the susceptibility-weighted imaging sequence, may lead to underestimation of CMB burden, particularly in lobar regions. Thus, this could influence both the classification of CMB regions and the strength of associations with ID risk. Second, given the scarcity of data regarding dementia diagnoses obtained through approaches other than expert adjudication (i.e., annual follow-up telephone interviews, informant interviews, and hospitalization and death certificate codes), we were unable to identify specific dementia subtypes and could not separately investigate the distinct relationships between MBs and Alzheimer’s disease or vascular dementia. Nevertheless, this solely restricts the stratified analyses since Alzheimer’s disease, whether occurring independently or alongside other neurological conditions, remains the predominant etiology for late-life dementia. Third, we excluded participants diagnosed with mild cognitive impairment at baseline. However, the primary findings did not change significantly in our sensitivity analyses, indicating that this may be less likely to introduce bias in the interpretation of the current results. Fourth, although we considered numerous confounders, residual confounding, such as chronic kidney disease, inflammatory markers, and lifestyle indices, may still have interfered with our findings. Despite these limitations, our study had some strengths, including the large number of participants and the high rate of follow-up. Fifth, while our findings indicated that the presence of MBs independently contributes to future dementia, we could not recommend specific interventions to reduce the risk of cognitive decline. A two-sample, two-step Mendelian Randomization study showed that the Sodium-glucose cotransporter 2 could reduce the risk of MBs but remains uncertain in reducing cognitive impairment [44,45].

## 5. Conclusions

In conclusion, both MB presence and MB severity at baseline were independently related to the long-term risk of ID in this large community-based elderly cohort. The strength of the association was found to be most significant among participants with mixed (lobar+subcortical) MBs, indicating the combined effect of CAA and hypertensive vasculopathy in dementia development. Furthermore, both lobar MB and superficial siderosis should be considered to understand the neurodegenerative contributions to cognitive decline. Collectively, our results provide amplified evidence and indicate that the presence of MBs or a high MB count, with some specificity for location, may be imperative for estimating ID risks.

## Supporting information

S1 Table Baseline characteristics of participants with and without incident dementia. Abbreviations: *APOE* = apolipoprotein E; HDL-C = high-density lipoprotein cholesterol; LDL-C = high-density lipoprotein cholesterol; MMSE = Mini Mental State Examination; WMH = white matter hyperintensity.
(DOCX)

S2 Table Characteristics of participants in 2011–2013 (Visit 5) by presence and number of microbleeds on brain MRI. Abbreviations: *APOE* = apolipoprotein E; HDL-C = high-density lipoprotein cholesterol; LDL-C = low-density lipoprotein cholesterol; MMSE = Mini Mental State Examination; WMH = white matter hyperintensity.
(DOCX)

S3 Table Sensitivity analysis on the association between microbleed patterns and incident dementia. Abbreviations: *APOE* = apolipoprotein E; MCI = mild cognitive impairment. ^a^ Hazard ratio of incident dementia in the population excluding those diagnosed with MCI, adjusted for age, sex, race, body mass index, depressive symptoms, *APOE* ε4 allele, educational level, ever smoking, HDL-C, LDL-C, hypertension, diabetes, and hippocampal volume. ^b^ Hazard ratio of incident dementia in the population excluding those whose dementia was ascertained in the first two years, adjusted for age, sex, race, body mass index, depressive symptoms, *APOE* ε4 allele, educational level, ever smoking, HDL-C, LDL-C, hypertension, diabetes, and hippocampal volume. ^c^ Hazard ratio of incident dementia in the population excluding participants with *APOE* ε4 status, adjusted for age, sex, race, body mass index, depressive symptoms, educational level, ever smoking, HDL-C, LDL-C, hypertension, diabetes, and hippocampal volume.
(DOCX)

S4 Table Sensitivity analysis on the association between microbleed patterns or superficial siderosis and incident dementia. Abbreviations: *APOE* = apolipoprotein E; MCI = mild cognitive impairment. ^a^ Hazard ratio of incident dementia in the population excluding those diagnosed with MCI, adjusted for age, sex, race, body mass index, depressive symptoms, *APOE* ε4 allele, educational level, ever smoking, HDL-C, LDL-C, hypertension, diabetes, and hippocampal volume. ^b^ Hazard ratio of incident dementia in the population excluding those whose dementia was ascertained in the first two years, adjusted for age, sex, race, body mass index, depressive symptoms, *APOE* ε4 allele, educational level, ever smoking, HDL-C, LDL-C, hypertension, diabetes, and hippocampal volume. ^c^ Hazard ratio of incident dementia in the population excluding participants with *APOE* ε4 status, adjusted for age, sex, race, body mass index, depressive symptoms, educational level, ever smoking, HDL-C, LDL-C, hypertension, diabetes, and hippocampal volume.
(DOCX)

S5 Table The net reclassification index and integrated discrimination improvement estimate of incident dementia. Abbreviations: CI = confidence interval; IDI = integrated discrimination improvement; LDL-C = low-density lipoprotein cholesterol; NRI = net reclassification index; Ref = reference. Basic model: Cox proportional-hazards model included age, sex, race, body mass index, depressive symptoms, *APOE* ε4 allele, educational level, ever smoking, HDL-C, LDL-C, hypertension, diabetes, and hippocampal volume.
(DOCX)

S1 Fig Flow diagram of study participants. (DOCX)

S2 Fig Graphic abstract. (PDF)
