# Supplementary material for: Cerebral microbleed patterns and the risk of incident dementia in elderly adults: The ARIC study
Source: PLoS One. 2026 Jan 21;21(1):e0340361. doi: 10.1371/journal.pone.0340361 (PMC12822971; doi:10.1371/journal.pone.0340361)
Supplement: S1 Table — Abbreviations: APOE = apolipoprotein E; HDL-C = high-density lipoprotein cholesterol; LDL-C = high-density lipoprotein cholesterol; MMSE = Mini Mental State Examination; WMH = white matter hyperintensity. (DOCX) [file pone.0340361.s001.docx]

**S1 Table. Baseline characteristics of participants with and without incident dementia.**

|  | Participants without incident dementia (*N* = 1142) | Participants with incident dementia (*N* = 390) |  |
| --- | --- | --- | --- |
| Age, years | 75.2 ± 4.9 | 78.5 ± 5.5 | <0.001 |
| Sex, % male | 462 (40.5) | 156 (40.0%) | 0.87 |
| Black, % | 286 (25.0) | 111 (28.5) | 0.18 |
| Body mass index, kg/m^2^ | 28.7 ± 5.5 | 27.9 ± 6.0 | 0.01 |
| Hypertension, % | 828 (72.5) | 306 (78.5) | 0.11 |
| Diabetes, % | 348 (30.5) | 136 (34.9) | 0.11 |
| Ever smoking, % | 619 (54.2) | 201 (51.5) | 0.36 |
| Education, % |  |  | <0.001 |
| < High school | 117 (10.2) | 72 (18.5) |  |
| High school | 477 (41.8) | 174 (44.6) |  |
| ≥ College | 548 (48.0) | 144 (36.9) |  |
| *APOE* ε4 status, % | 283 (24.8) | 140 (35.9) | <0.001 |
| HDL-C, mmol/L | 1.4 ± 0.4 | 1.4 ± 0.3 | 0.73 |
| LDL-C, mmol/L | 2.7 ± 0.9 | 2.7 ± 0.9 | 0.54 |
| MMSE score | 28 (27-29) | 27 (25-28) | <0.001 |
| Depressive symptoms | 59 (5.1) | 43 (11.0) | <0.001 |
| MRI characteristics |  |  |  |
| Hippocampus volume, mL | 7.1 ± 1.0 | 6.5 ± 1.0 | <0.001 |
| Nonhippocampal AD signature region volume, mL | 53.2 ± 6.2 | 50.8 ± 6.3 | <0.001 |
| WMH volume, mL | 10.0 (5.7-18.2) | 16.7 (9.2-31.5) | <0.001 |
| Lacunar infarcts, % | 163 (14.3) | 96 (24.6) | <0.001 |
| Microbleeds, % | 231 (20.2) | 122 (31.3) | <0.001 |

Abbreviations: *APOE* = apolipoprotein E; HDL-C = high-density lipoprotein cholesterol; LDL-C = high-density lipoprotein cholesterol; MMSE = Mini Mental State Examination; WMH = white matter hyperintensity.
