# Supplementary material for: Cerebral microbleed patterns and the risk of incident dementia in elderly adults: The ARIC study
Source: PLoS One. 2026 Jan 21;21(1):e0340361. doi: 10.1371/journal.pone.0340361 (PMC12822971; doi:10.1371/journal.pone.0340361)
Supplement: S2 Table — Abbreviations: APOE = apolipoprotein E; HDL-C = high-density lipoprotein cholesterol; LDL-C = low-density lipoprotein cholesterol; MMSE = Mini Mental State Examination; WMH = white matter hyperintensity. (DOCX) [file pone.0340361.s002.docx]

**S2 Table.** **Characteristics of participants in 2011–2013 (Visit 5) by presence and number of microbleeds on brain MRI.**

|  | No microbleeds  (*N* = 1179) | 1 microbleed  (*N* = 210) | ≥ 2 microbleeds  (*N* = 143) |  |
| --- | --- | --- | --- | --- |
| Age, years | 75.8 ± 5.2 | 76.5 ± 5.3 | 77.5 ± 5.5 | <0.001 |
| Sex, % male | 459 (38.9) | 91 (43.3%) | 68 (47.6%) | 0.01 |
| Black, % | 296 (25.1) | 60 (28.6) | 41 (28.7) | 0.42 |
| Body mass index, kg/m^2^ | 28.6 ± 5.7 | 28.0 ± 5.1 | 28.3 ± 6.0 | 0.51 |
| Hypertension, % | 866 (73.5) | 159 (75.7) | 109 (76.2) | 0.65 |
| Diabetes, % | 377 (32.0) | 62 (29.5) | 45 (31.5) | 0.78 |
| Ever smoking, % | 623 (52.8) | 122 (58.1) | 75 (52.5) | 0.36 |
| Education, % |  |  |  | 0.60 |
| < High school | 139 (11.8) | 27 (12.9) | 23 (16.1) |  |
| High school | 499 (42.0) | 93 (44.3) | 59 (41.3) |  |
| ≥ College | 541 (45.9) | 90 (42.9) | 61 (42.7) |  |
| *APOE* ε4 status, % | 318 (27.0) | 61 (29.1) | 44 (30.8) |  |
| HDL-C, mmol/L | 1.4 ± 0.4 | 1.4 ± 0.3 | 1.3 ± 0.3 | 0.10 |
| LDL-C, mmol/L | 2.8 ± 0.9 | 2.7 ± 0.9 | 2.7 ± 0.9 | 0.47 |
| MMSE score | 28 (27-29) | 28 (26-29) | 28 (26-29) | 0.02 |
| Depressive symptoms | 73 (6.2) | 13 (6.2) | 16 (11.2) | 0.07 |
| MRI characteristics |  |  |  |  |
| Hippocampus volume, mL | 6.9 ± 1.0 | 6.9 ± 1.0 | 6.8 ± 1.0 | 0.07 |
| Nonhippocampal AD signature  region volume, mL | 52.7 ± 6.2 | 52.3 ± 6.3 | 51.8 ± 6.4 | 0.22 |
| WMH volume, mL | 10.7 (5.9-18.8) | 11.8 (6.8-23.4) | 20.2 (10.1-34.5) | <0.001 |
| Lacunar infarcts, % | 170 (14.4) | 42 (20.0) | 47 (32.9) | <0.001 |

Abbreviations: *APOE* = apolipoprotein E; HDL-C = high-density lipoprotein cholesterol; LDL-C = low-density lipoprotein cholesterol; MMSE = Mini Mental State Examination; WMH = white matter hyperintensity.
