# Supplementary material for: Cerebral microbleed patterns and the risk of incident dementia in elderly adults: The ARIC study
Source: PLoS One. 2026 Jan 21;21(1):e0340361. doi: 10.1371/journal.pone.0340361 (PMC12822971; doi:10.1371/journal.pone.0340361)
Supplement: S4 Table — Abbreviations: APOE = apolipoprotein E; MCI = mild cognitive impairment. a Hazard ratio of incident dementia in the population excluding those diagnosed with MCI, adjusted for age, sex, race, body mass index, depressive symptoms, APOE ε4 allele, educational level, ever smoking, HDL-C, LDL-C, hypertension, diabetes, and hippocampal volume. b Hazard ratio of incident dementia in the population excluding those whose dementia was ascertained in the first two years, adjusted for age, sex, race, body mass index, depressive symptoms, APOE ε4 allele, educational level, ever smoking, HDL-C, LDL-C, hypertension, diabetes, and hippocampal volume. c Hazard ratio of incident dementia in the population excluding participants with APOE ε4 status, adjusted for age, sex, race, body mass index, depressive symptoms, educational level, ever smoking, HDL-C, LDL-C, hypertension, diabetes, and hippocampal volume. (DOCX) [file pone.0340361.s004.docx]

**S4 Table.** **Sensitivity analysis on the association between microbleed patterns or superficial siderosis and** **incident dementia.**

| Microbleed patterns or superficial siderosis | Excluding participants diagnosed with MCI^a^ | | |  | Excluding participants whose dementia was ascertained in the first two years^b^ | | |  | Excluding participants with *APOE* ε4 status^c^ | | |
| --- | --- | --- | --- | --- | --- | --- | --- | --- | --- | --- | --- |
|  | HR 95%CI | | p value |  | HR 95%CI | p value | |  | HR 95%CI | p value | |
| No microbleed or superficial siderosis | 1 (reference) |  | |  | 1 (reference) | |  |  | 1 (reference) | |  |
| Only subcortical microbleeds | 1.35 (0.88-2.08) | 0.17 | |  | 1.25 (0.93-1.68) | | 0.13 |  | 1.31 (0.93-1.85) | | 0.12 |
| Only lobar microbleeds or superficial siderosis | 1.62 (1.15-2.29) | 0.006 | |  | 1.82 (1.16-2.86) | | 0.009 |  | 1.93 (1.06-3.52) | | 0.03 |
| Mixed(subcortical microbleeds + either lobar microbleeds or superficial siderosis) | 2.17 (1.22-3.87) | | 0.009 |  | 1.93 (1.34-2.78) | | <0.001 |  | 2.32 (1.46-3.67) | | <0.001 |

Abbreviations: *APOE* = apolipoprotein E; MCI = mild cognitive impairment.

^a^ Hazard ratio of incident dementia in the population excluding those diagnosed with MCI, adjusted for age, sex, race, body mass index, depressive symptoms, *APOE* ε4 allele, educational level, ever smoking, HDL-C, LDL-C, hypertension, diabetes, and hippocampal volume.

^b^ Hazard ratio of incident dementia in the population excluding those whose dementia was ascertained in the first two years, adjusted for age, sex, race, body mass index, depressive symptoms, *APOE* ε4 allele, educational level, ever smoking, HDL-C, LDL-C, hypertension, diabetes, and hippocampal volume.

^c^ Hazard ratio of incident dementia in the population excluding participants with *APOE* ε4 status, adjusted for age, sex, race, body mass index, depressive symptoms, educational level, ever smoking, HDL-C, LDL-C, hypertension, diabetes, and hippocampal volume.
