# Supplementary material for: Cerebral microbleed patterns and the risk of incident dementia in elderly adults: The ARIC study
Source: PLoS One. 2026 Jan 21;21(1):e0340361. doi: 10.1371/journal.pone.0340361 (PMC12822971; doi:10.1371/journal.pone.0340361)
Supplement: S5 Table — Abbreviations: CI = confidence interval; IDI = integrated discrimination improvement; LDL-C = low-density lipoprotein cholesterol; NRI = net reclassification index; Ref = reference. Basic model: Cox proportional-hazards model included age, sex, race, body mass index, depressive symptoms, APOE ε4 allele, educational level, ever smoking, HDL-C, LDL-C, hypertension, diabetes, and hippocampal volume. (DOCX) [file pone.0340361.s005.docx]

**S5 Table. The net reclassification index and integrated discrimination improvement estimate of incident dementia.**

| Variables | NRI | | IDI | |
| --- | --- | --- | --- | --- |
|  | Estimate (95% CI), % | p Value | Estimate (95% CI), % | p Value |
| Basic model | Ref |  | Ref |  |
| Basic model + Any microbleeds | 17.00 (8.40–24.40) | < 0.001 | 3.00 (1.50–4.70) | < 0.001 |
| Basic model + Any lobar microbleeds | 16.20 (7.10–25.40) | < 0.001 | 3.00 (1.10–5.10) | < 0.001 |
| Basic model + Any subcortical microbleeds | 17.20 (8.30–24.30) | < 0.001 | 3.10 (1.40–4.90) | < 0.001 |
| Basic model + Only lobar microbleeds | 17.80 (8.60–26.50) | < 0.001 | 3.00 (1.40–5.40) | < 0.001 |
| Basic model + Only subcortical microbleeds | 17.20 (9.70–26.00) | < 0.001 | 3.20 (1.70–5.20) | < 0.001 |
| Basic model + Mixed (subcortical+lobar microbleeds) | 15.60 (6.70–23.50) | = 0.007 | 2.90 (1.20–5.40) | < 0.001 |

Abbreviations: CI = confidence interval; IDI = integrated discrimination improvement; LDL-C = low-density lipoprotein cholesterol; NRI = net reclassification index; Ref = reference.

Basic model: Cox proportional-hazards model included age, sex, race, body mass index, depressive symptoms, *APOE* ε4 allele, educational level, ever smoking, HDL-C, LDL-C, hypertension, diabetes, and hippocampal volume.
