# Supplementary figures and images for: Cerebral microbleed patterns and the risk of incident dementia in elderly adults: The ARIC study
Source: PLoS One. 2026 Jan 21;21(1):e0340361. doi: 10.1371/journal.pone.0340361 (PMC12822971; doi:10.1371/journal.pone.0340361)

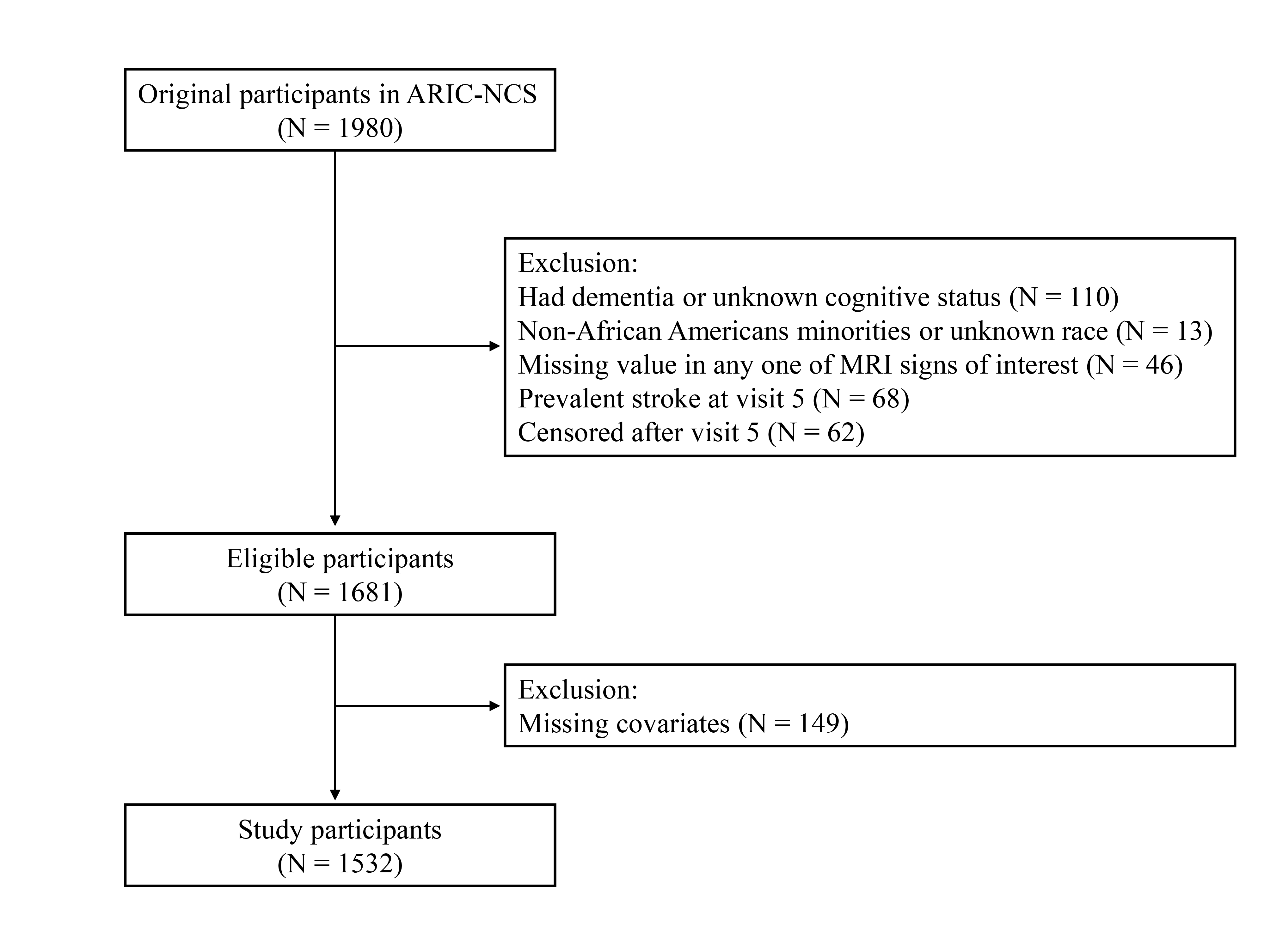


**S1 Fig. Flow diagram of study participants.**

Supplement: S1 Fig — (DOCX) [file pone.0340361.s006.docx]
