# Supplementary material for: Cerebral microbleed patterns and the risk of incident dementia in elderly adults: The ARIC study
Source: PLoS One. 2026 Jan 21;21(1):e0340361. doi: 10.1371/journal.pone.0340361 (PMC12822971; doi:10.1371/journal.pone.0340361)
Supplement: S2 Fig — (PDF) [file pone.0340361.s007.pdf]

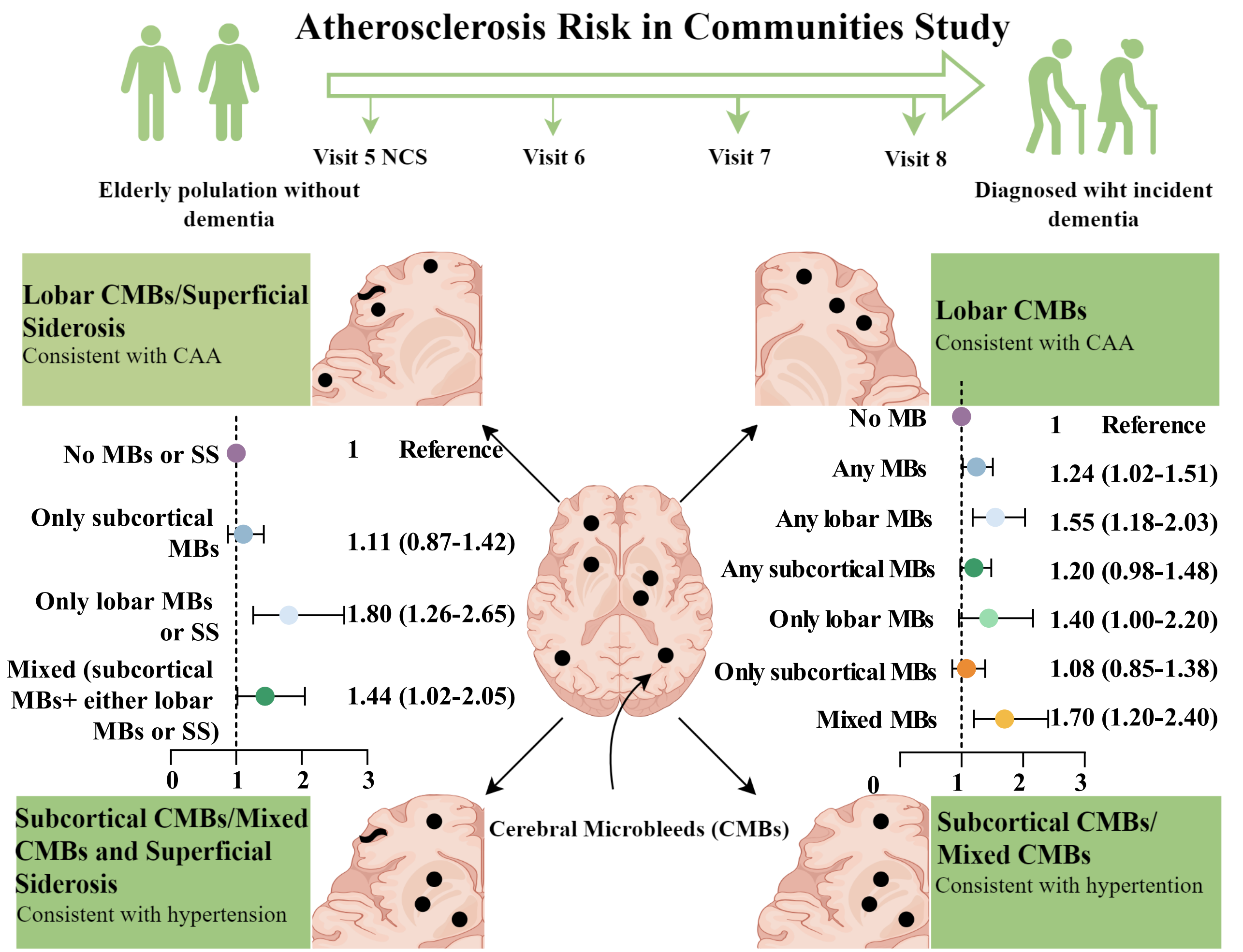

Key Findings

In this large community-based elderly cohort, we identified that the presence of MBs or a high MB count (i.e., ≥ 2), with some specificity for location, was independently associated with an increased risk of incident dementia over a 9-year follow-up.

Abbreviations

CAA = Cerebral amyloid angiopathy; CMBs = Cerebral microbleeds; MB = Microbleeds; SS = superficial siderosis.
